# Effect of a 5:2 intermittent fasting diet on obese patients with polycystic ovary syndrome

**DOI:** 10.3389/fendo.2026.1758805

**Published:** 2026-03-18

**Authors:** Wencheng An, Fang Chen, Bingyan Wang, Shujing Zhou, Lin Jiang, Bo An, Zhijun Cui, Xiaohong Ou, Wei Huang, Huixian Yan

**Affiliations:** 1 Department of Endocrinology, Beijing Haidian Hospital, Beijing Haidian Section of Peking University Third Hospital, Haidian, Beijing, China; 2 Department of General Surgery, Peking University Third Hospital, Haidian, Beijing, China

**Keywords:** 5:2 intermittent fasting diet, diet, obesity, polycystic ovary syndrome, weight loss

## Abstract

**Introduction:**

Polycystic ovary syndrome (PCOS) is a common endocrine disorder that affects fertility and metabolism. Obesity, present in a significant proportion of PCOS patients, exacerbates insulin resistance (IR) and worsens reproductive outcomes. This study explores the effects of a 12-week 5:2 intermittent fasting diet with meal replacement (MR) on body weight, metabolic, and endocrine markers in obese women with PCOS.

**Methods:**

A non-randomized, single-arm interventional study was conducted with 90 obese women diagnosed with PCOS. Participants were assigned to three groups: 5:2 intermittent fasting with MR, non-meal replacement intermittent fasting, and no intervention. To reduce potential confounding, the study utilized Propensity Score Matching (PSM) to match baseline characteristics such as age, body mass index (BMI), and metabolic markers. Primary outcomes included changes in body weight, BMI, waist circumference, fasting insulin, glucose levels, and reproductive hormone markers. Menstrual regularity and ovulation frequency were also assessed.

**Results:**

The intervention group (5:2 intermittent fasting with MR) showed significant reductions in body weight (from 77.15 kg to 69.1 kg, P < 0.001), waist circumference, and BMI. Insulin resistance (HOMA-IR) decreased significantly (P = 0.008), and a reduction in 2-hour glucose levels (P = 0.025) was observed. Although metabolic markers improved, reproductive hormones (follicle-stimulating hormone [FSH], luteinizing hormone [LH], estradiol, progesterone) did not show significant changes. However, menstrual regularity improved in 80% of participants, and 50% showed improved ovulation frequency. The non-meal replacement intermittent fasting group also showed improvements, but to a lesser extent.

**Conclusions:**

The 5:2 intermittent fasting diet with MR significantly reduces body weight, improves insulin sensitivity, and enhances metabolic markers in obese women with PCOS. Although reproductive hormone levels did not significantly change, menstrual and ovulatory function improved. Future studies with larger sample sizes and longer follow-up are needed to explore the long-term effects of intermittent fasting on reproductive health in PCOS patients.

## 1 Introduction

Polycystic ovary syndrome (PCOS) is a syndrome characterized by androgen hyperplasia, anovulation, and polycystic ovary morphology, and patients with PCOS are prone to menstrual disorders, infertility, insulin resistance (IR), and metabolic disorders (1–3). Obesity exists in 30–60% of PCOS patients, which further aggravates IR and affects oocyte and embryo quality, thus considerably reducing the average fertilization rate, clinical pregnancy rate, and live birth rate and considerably increasing the abortion rate (4). Lifestyle improvement and lifestyle intervention have become the consensus for PCOS treatment and are listed as the first-line treatment for PCOS (5). For overweight and obese PCOS patients, a weight loss of 5–10% will help improve reproductive and metabolic indicators while promoting mental health (6).

Several recent studies have shown that intermittent fasting has good short-term and long-term effects on obesity control (7, 8). For women with PCOS, intermittent fasting is shown to be an effective lifestyle intervention as it targets hyperandrogenism, insulin resistance, and menstrual irregularities (9, 10). However, a systematic review assessing the studies on the impact of intermittent fasting on anthropometric measures and glycemic control, lipid profile, hormonal, and oxidative stress, and inflammatory markers, reported mixed results (11). However, the literature on specific methodologies of intermittent fasting remains scant.

The 5:2 intermittent fasting diet is one of the most commonly used plans, consisting of two nonconsecutive fasting days and five habitual intake days per week (12). An intermittent fasting strategy is usually administered with meal replacement (MR). MR refers to a prepacked food or beverage that can replace one or more meals to provide a nutritional supply. Previous studies have shown that MR can significantly reduce patients’ body weight and alleviate diabetes (13). Some studies have confirmed that compared with continuous energy-restricted diets, the 5:2 intermittent fasting diet with MR has a better effect on body weight, Hemoglobin (HbA1c), and related indicators in patients with type 2 diabetes (14, 15).

MR on fasting days is commonly used in diabetes and obesity trials, but has rarely been studied in PCOS. As the studies on the effect of the 5:2 intermittent fasting diet strategy for the treatment of obese patients with PCOS are still limited, the objective of this study was to investigate the effects of a 12-week 5:2 intermittent fasting diet with the MR strategy for improving body weight, metabolism, and endocrine markers in obese patients with PCOS.

## 2 Materials and methods

### 2.1 Study design and sampling

This interventional study included obese PCOS patients admitted to Beijing Haidian Hospital (Beijing, China) from May 15, 2021 to October 3, 2023. Ethical approval for this study was obtained from the Medical Ethics Committee of Beijing Haidian Hospital (BHHMEC-XM-2021-21). Informed consent was obtained from all subjects. The diagnosis of PCOS was based on Rotterdam diagnostic criteria, including the following criteria: 1) oligo- or anovulation, 2) clinical and/or biochemical signs of hyperandrogenism, and 3) polycystic ovaries and exclusion of other etiologies (congenital adrenal hyperplasia, androgen-secreting tumors, Cushing’s syndrome). PCOS is diagnosed when at least two of the three criteria are fulfilled (16).

The patients included in this study met the following criteria: 1) age 20–45 years old; 2) confirmed PCOS; 3) Body Mass Index (BMI) ≥ 24 kg/m2; 4) the patient has not taken sex hormones or drugs that affect sugar and lipid metabolism in the previous week, and the patient was required to not use these drugs within 12 weeks of light fasting treatment; and 5).

Patients with Cushing’s syndrome, congenital adrenal hyperplasia, and other endocrine diseases, those with adrenal or ovarian tumors, those with vital organ diseases, those who had poor compliance, and those who were pregnant and lactating or received sex hormones or drugs that affect glucose and lipid metabolism during the observation period were excluded.

### 2.2 Propensity score matching method to establish control groups

In order to better assess the effects of the 5:2 intermittent fasting diet and reduce potential confounding factors, this study utilized the PSM method. By calculating propensity scores based on baseline characteristics such as age, BMI, fasting glucose, and hormone levels, the intervention group was matched with control groups. By calculating the patients’ propensity scores, we matched the intervention group (5:2 intermittent fasting with meal replacement) with two control groups. The propensity scores were calculated using the following baseline characteristics: age, body weight (kg), BMI, fasting blood glucose (FPG), 2-hour oral glucose tolerance test (OGTT) blood glucose (Glu2), hormone levels (such as LH, FSH, free testosterone), and insulin resistance index (HOMA-IR). Through propensity score matching, we ensured that the intervention group and the control groups were similar in these baseline characteristics, thereby reducing the impact of selection bias and making the evaluation of the intervention effects more reliable.

The control groups in this study included: 1) No Treatment Group: Patients in this group did not receive any intervention, serving as a control for natural fluctuations in body weight and metabolic markers over time. This group helped establish baseline measurements, accounting for changes that may occur naturally without any treatment or dietary intervention. 2) Non-Meal Replacement Intermittent Fasting Group: Patients in this group followed the same 5:2 intermittent fasting diet plan as the intervention group but did not use meal replacements. This design allowed us to evaluate the specific impact of meal replacements, compared to regular food intake, on weight loss and metabolic markers such as insulin resistance and blood glucose levels. The comparison between this group and the intervention group helped isolate the effect of meal replacement products as part of the 5:2 fasting regimen.

### 2.3 Interventions

In this study, a 5:2 intermittent fasting diet was adopted as the intervention. MR food was consumed for discontinuous 2 days per week with a daily energy intake of 500 kcal, and on the remaining 5 days, the patients were free to eat according to personal eating habits (17). The recommended diet plan for the MR days was as follows: for breakfast, an egg and MR product A (Kang zhijun cereal and fruit meal pack No. 2, 20 g/pack, three packs, Beijing Metabolic Control Technology Co., Ltd. Beijing, China, **Supplementary Table 1**); for lunch, 100 g of low-sugar fruit (such as cucumbers, tomatoes, and oranges) and MR product A; for dinner, 200–400 g of green leafy vegetables (such as spinach and cabbage) and MR product A. The total daily energy intake was 500 kcal. On the remaining 5 days of the week, breakfast and lunch were typical meals (less oil and sugar are recommended), with dinner including MR product B (Kang zhijun cereal fruit and vegetable meal pack No. 5, 20 g/pack, one pack, Beijing MetabolicControl Technology Co., Ltd., **Supplementary Table 1**) instead of staple food and other regular diets. The intervention lasted for 12 weeks, during which participants were asked to record their daily diet in detail. To ensure accurate reporting and minimize potential bias from self-reported data, the research team implemented digital tracking tools and conducted regular dietary audits. These methods allowed for continuous monitoring of adherence to the diet plan, ensuring that participants followed the prescribed dietary regimen. Patients who could not strictly follow the 5:2 intermittent fasting diet plan, as determined through these adherence checks, were removed from the study.

### 2.4 Outcomes

This study’s primary outcomes were changes in body weight, BMI, and hip circumference. Endocrine indexes included the HbA1c level, FPG level, fasting insulin (FIN) level, fasting C-peptide level, lipid profiles (total cholesterol, triglycerides, high-density lipoprotein cholesterol [HDL-C], and low-density lipoprotein cholesterol levels), uric acid levels, and homeostasis model assessment of insulin resistance (HOMA-IR = FPG [mmol/L] × fasting insulin [μU/mL]/22.5). The OGTT results included FPG and the 2-h blood glucose (Glu2) after oral administration of 75 g of glucose. Reproductive hormone indicators were also tested, including follicle-stimulating hormone (FSH), luteinizing hormone (LH), free testosterone, estradiol, prolactin, and progesterone levels. In addition to hormonal measurements, menstrual cycle regularity and ovulation frequency were also assessed before and after the intervention. Menstrual status was recorded by the participants, including the number of menstrual cycles over a 3-month period prior to and after the intervention. Ovulation status was determined by tracking luteal phase progesterone levels or by using ovulation test kits. The baseline was measured 1 day before the intervention. The outcomes were evaluated and re-evaluated at the end of the intervention (12 weeks). The laboratory department of Beijing Haidian Hospital performed all laboratory tests.

### 2.5 Statistical analysis

SPSS17.0 statistical software was used to process the data. The measurement data were expressed as (Mean ± Standard Deviation), and a paired *t*-test was used before and after the intervention. Counting data are expressed in relative numbers. P < 0.05 was considered statistically significant.

## 3 Results

### 3.1 Baseline characteristics

A total of 90 patients who met the inclusion criteria participated in this study: 40 in the intervention group (5:2 intermittent fasting + meal replacement), 30 in the no intervention group, and 30 in the non-meal replacement intermittent fasting group. After 12 weeks of intervention, all 90 patients completed follow-up, with 10 patients from the intervention group (5:2 intermittent fasting + meal replacement) leaving due to inability to adhere to or return for follow-up.

**Table 1** presents the changes in baseline characteristics and post-intervention outcomes for the intervention group, no intervention group, and non-meal replacement intermittent fasting group. Overall, the intervention group (5:2 intermittent fasting + meal replacement) showed significant improvements in body weight and insulin resistance index (HOMA-IR), with body weight decreasing from 77.15 kg to 69.1 kg (P = 0.017) and a significant decrease in HOMA-IR (P = 0.008). In contrast, the no intervention group and the non-meal replacement intermittent fasting group showed smaller changes in body weight and metabolic markers, and these differences did not reach statistical significance.

**Table 1:** Baseline characteristics and post-intervention changes in each group.

Index	Intervention group (5:2 Intermittent Fasting + Meal Replacement, n=30)	No intervention group (n=30)	Non-meal replacement intermittent fasting group (n=30)	P value
Age (years)	29(25–32)	30(28–33)	29(26–32)	
BMI(kg/m²)				
Before Intervention	29.64 ± 7.24	30.20 ± 6.90	28.90 ± 6.50	0.561
After Intervention	26.14 ± 7.66	28.50 ± 6.90	27.50 ± 6.30	0.055
Body weight(kg)				
Before Intervention	77.15(70.00–89.70)	78.30(71.20–85.00)	76.80(69.50–83.00)	0.804
After Intervention	69.10(61.70–76.90)	77.00(70.50–83.50)	74.50(67.00–81.00)	<0.001
FPG(mmol/L)				
Before Intervention	5.53 ± 1.26	5.60 ± 1.15	5.50 ± 1.10	0.734
After Intervention	5.30 ± 1.12	5.60 ± 1.18	5.47 ± 1.05	0.622
2-h blood glucose(Glu2, mmol/L)				
Before Intervention	5.98(5.29–10.83)	6.00(5.50–10.00)	5.85(5.30–9.50)	0.718
After Intervention	5.60(5.10–9.50)	6.10(5.60–10.20)	5.70(5.30–9.00)	0.403
HbA1c (%)				
Before Intervention	5.69(5.44–6.06)	5.75(5.50–6.20)	5.65(5.40–6.00)	0.108
After Intervention	5.67(5.25–5.91)	5.70(5.50–6.00)	5.60(5.35–5.80)	0.080
HOMA-IR				
Before Intervention	5.69 ± 5.33	5.80 ± 5.00	5.40 ± 5.10	0.877
After Intervention	3.80 ± 4.56	5.70 ± 5.20	4.50 ± 4.90	0.008

FPG, fasting plasma glucose; HbA1c, hemoglobin A1c; HOMA-IR, homeostasis model assessment of insulin resistance.

Post-intervention, the intervention group also showed improvements in BMI, fasting blood glucose, HbA1c, and OGTT blood glucose, although the changes in fasting blood glucose and OGTT blood glucose were not statistically significant. In comparison, the changes in these markers were smaller in the other two groups, particularly in the no intervention group.

### 3.2 Main outcomes

**Figure 1:**
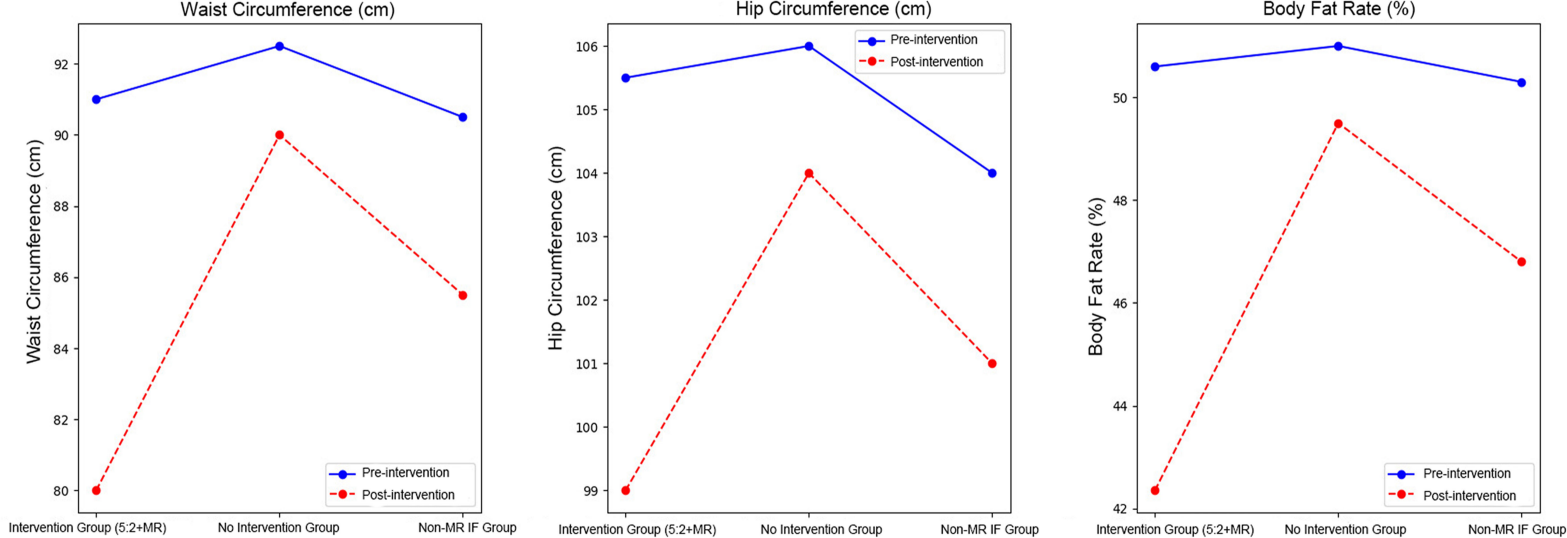
Changes in body composition indicators (waist circumference, hip circumference, and body fat rate) before and after the 5:2 intermittent fasting intervention with meal replacement (MR) in each group. 5:2 intermittent fasting diet, intermittent fasting plan consisting of 2 non-consecutive fasting days and 5 days of typical intake and a meal replacement diet per week.

The changes in waist circumference, hip circumference, and body fat rate before and after the intervention are summarized in **Figure 1**. The intervention group (5:2 intermittent fasting with meal replacement) showed significant reductions in waist circumference, hip circumference, and body fat rate compared to the other groups. Specifically, the waist circumference and body fat rate in the intervention group decreased significantly, while the non-meal-replacement intermittent fasting group and the no intervention group showed smaller changes. The P values for these changes were <0.001 for waist circumference, 0.01 for hip circumference, and <0.001 for body fat rate, indicating significant differences in the intervention group.

### 3.3 Changes in biochemical markers and hormone levels

**Table 2** presents the changes in biochemical markers and hormone levels before and after the 5:2 intermittent fasting intervention with MR in the intervention group, no intervention group, and non-meal-replacement intermittent fasting group. Additionally, menstrual regularity and ovulation frequency were tracked. Prior to the intervention, 65% of participants in the intervention group had irregular menstrual cycles, and 40% reported regular ovulation. After the 12-week intervention, 80% of participants in the intervention group reported regular menstrual cycles, and 50% experienced improved ovulatory function. Similar trends were observed in the Non-Meal Replacement Intermittent Fasting Group, where menstrual regularity and ovulation rates improved, although to a lesser extent compared to the intervention group. These changes in menstrual and ovulatory function align with improvements in metabolic and endocrine markers observed post-intervention.

**Table 2:** Changes in biochemical markers and hormone levels.

Index	Intervention group (5:2 Intermittent Fasting + Meal Replacement, n=30)	No intervention group (n=30)	Non-meal replacement intermittent fasting group (n=30)	P value
FIN (μmol/L)				
Before Intervention	18.45(12.03–27.38)	19.10(13.00–28.00)	18.70(12.50–26.50)	0.002
After Intervention	10.78(7.45–14.75)	17.00(12.00–25.00)	14.50(10.00–20.00)	<0.001
Glu2 (mmol/L)				
Before Intervention	5.98(5.29–10.83)	6.10(5.40–10.00)	5.95(5.20–9.90)	0.025
After Intervention	5.63(5.21–6.54)	6.00(5.50–10.00)	5.75(5.30–8.80)	0.032
TC (mmol/L)				
Before Intervention	5.14 ± 0.88	5.10 ± 0.75	5.20 ± 0.85	0.439
After Intervention	4.87 ± 0.90	5.05 ± 0.95	4.90 ± 0.80	0.064
Triglycerides (mmol/L)				
Before Intervention	1.99 ± 1.61	2.10 ± 1.50	2.00 ± 1.50	0.115
After Intervention	1.35 ± 0.55	1.80 ± 0.70	1.50 ± 0.65	<0.001
LDL-C (mmol/L)				
Before Intervention	3.33 ± 0.69	3.40 ± 0.75	3.25 ± 0.70	0.55
After Intervention	3.15 ± 0.79	3.30 ± 0.85	3.20 ± 0.75	0.192
HDL-C (mmol/L)				
Before Intervention	1.24 ± 0.27	1.25 ± 0.30	1.23 ± 0.25	0.796
After Intervention	1.25 ± 0.35	1.22 ± 0.32	1.28 ± 0.30	0.517
UA (μmol/L)				
Before Intervention	369.00(329.75–454.75)	370.00(340.00–460.00)	365.00(320.00–450.00)	0.281
After Intervention	356.00(295.00–426.00)	368.00(340.00–455.00)	360.00(320.00–440.00)	0.015
FSH (IU/L)				
Before Intervention	5.13(3.66–6.60)	5.20(4.00–6.50)	5.10(4.00–6.00)	0.93
After Intervention	5.07(4.08–5.97)	5.10(4.50–5.90)	5.05(4.20–5.80)	0.823
LH (IU/L)				
Before Intervention	8.29(4.56–13.74)	8.50(5.00–12.00)	8.00(4.50–12.50)	0.115
After Intervention	5.70(3.89–11.26)	6.00(4.50–10.50)	5.90(4.00–9.00)	0.022
Prolactin (ng/mL)				
Before Intervention	11.66(9.31–17.15)	11.80(9.00–17.50)	11.50(9.10–16.00)	0.143
After Intervention	15.49(10.84–21.02)	15.00(11.00–19.00)	15.30(11.50–20.00)	0.034
Estradiol (pg/mL)				
Before Intervention	39.00(32.75–65.65)	38.00(33.00–63.00)	39.50(32.00–62.00)	0.133
After Intervention	31.90(25.89–33.05)	32.50(28.00–35.00)	32.00(27.00–34.50)	0.04
Free testosterone (ng/mL)				
Before Intervention	0.62(0.47–0.79)	0.60(0.50–0.80)	0.61(0.48–0.75)	0.099
After Intervention	0.53(0.41–0.65)	0.58(0.45–0.70)	0.54(0.42–0.68)	0.028
Progesterone (ng/mL)				
Before Intervention	0.069(0.050–0.182)	0.070(0.050–0.180)	0.070(0.050–0.175)	0.292
After Intervention	0.160(0.050–0.262)	0.160(0.050–0.250)	0.150(0.050–0.240)	0.02

5:2 intermittent fasting diet, intermittent fasting plan consisting of 2 non-consecutive fasting days and 5 days of typical intake and a meal replacement diet per week; FIN, fasting insulin; Glu2, 2-h blood glucose after an oral glucose tolerance test; TC, total cholesterol; LDL-C, low-density lipoprotein cholesterol; HDL-C, high-density lipoprotein cholesterol; UA, uric acid; FSH, follicle-stimulating hormone; LH, luteinizing hormone.

The intervention group showed significant improvements, including a marked decrease in FIN (μmol/L) (P < 0.001) and a reduction in Glu2 (mmol/L) (P = 0.025). Triglycerides (mmol/L) also decreased significantly (P < 0.001), while LDL-C (mmol/L) and HDL-C (mmol/L) exhibited minor changes without significant differences between groups. Furthermore, LH (IU/L) and Prolactin (ng/mL) levels showed significant changes (P = 0.022 and P = 0.034, respectively) in the intervention group post-intervention. Although hormonal markers like FSH, Estradiol, and Progesterone showed some changes, they were not statistically significant.

## 4 Discussion

PCOS is a common reproductive endocrine and metabolic disease that seriously affects the quality of life, fertility, and long-term health of patients (18). Lifestyle intervention, such as diet control and exercise, is the primary treatment for overweight or obese PCOS patients, which can effectively improve patients’ health-related quality of life (19).

The light fasting mode, also known as the intermittent fasting 5:2 mode, is an effective medical weight loss method recognized internationally. It can control weight, the lipid metabolism index, and the insulin resistance level (20) and enhance the treatment benefits of diabetes, cardiovascular and cerebrovascular diseases, and other chronic diseases (21). Previous studies found that after an intervention of daily fasting for 8 weeks, patients’ body weights decreased by 5.6 ± 1.0 kg on average, their waist circumferences decreased by 4.0 cm on average, and body fat content and TC, LDL-C, and TG concentrations all decreased (22). A meta-analysis showed that light fasting can effectively reduce weight, prevent type 2 diabetes, and improve blood sugar, insulin, LDL-C, HDL-C, and other metabolic markers in overweight and obese patients (23). Current studies on weight loss in the PCOS population primarily focus on the nutrition program of calorie-restricted diets (CRDs). Previous studies found that after CRD treatment, PCOS patients’ body weights, body fat, and visceral fat area (VFA) decreased, and lipid metabolism disorders and insulin resistance improved (24). However, the effect of a 5:2 intermittent fasting diet with MR on overweight and obese PCOS patients has not been studied in China. Thus, we focused on the effect of a 5:2 intermittent fasting diet on improving body weight and endocrine indexes in obese PCOS patients. Unlike most prior PCOS diet trials that use continuous daily calorie restriction or other intermittent fasting patterns, this protocol prescribed two non-consecutive fasting days per week at 500 kcal (using specified meal‐replacement foods) and five days of a usual diet.

Our findings show significant improvements in weight and insulin metabolism following the 5:2 intermittent fasting diet. At the 12-week follow-up, the average body weight and waist circumference significantly decreased. Fasting insulin (P = 0.002) and insulin resistance (P = 0.017) were also reduced, along with a reduction in 2-hour OGTT glucose (P = 0.025). These findings are consistent with recent meta-analyses showing that intermittent fasting in PCOS reliably lowers weight, BMI, fasting glucose, and insulin resistance (25). However, despite these improvements, we did not observe significant changes in lipid panels or reproductive hormones, which may be attributed to several factors.

One critical aspect that warrants attention is the lack of significant changes in reproductive hormones, despite the expected impact of the 5:2 intermittent fasting diet on this key area of PCOS pathology. The hormonal regulation of reproductive functions is central to PCOS, and interventions that affect metabolic markers like insulin resistance often lead to improvements in reproductive outcomes as well. In this study, however, while insulin and metabolic markers showed improvement, many of the reproductive hormones, including FSH, LH, and estradiol, did not demonstrate statistically significant changes post-intervention. The lack of change in reproductive hormones could be due to several factors. 1) Duration of the Intervention: A 12-week intervention may not be long enough to produce noticeable changes in reproductive markers. Hormonal regulation, particularly in PCOS, is complex and may require more extended periods of intervention to observe substantial changes. 2) Sample Size and Variability: Given the relatively small sample size of 30 participants in the intervention group, the study may have lacked sufficient statistical power to detect subtle changes in reproductive hormones. Larger studies may be needed to better capture the nuanced effects on reproductive health. 3) Effect of Fasting on Hormonal Regulation: While intermittent fasting has been shown to influence insulin and metabolic pathways, its direct effect on reproductive hormones in women with PCOS may be more limited or take longer to manifest. Previous studies have suggested that lifestyle interventions like weight loss and insulin-sensitizing treatments can improve reproductive outcomes, but these effects may depend on a combination of factors beyond metabolic improvements alone (5). It is possible that the 5:2 fasting diet, despite improving insulin sensitivity, may not be sufficient by itself to induce significant changes in reproductive hormones without additional interventions targeting androgen excess or ovulatory function. 4) Impact of Free Eating Days: The variability introduced by the five days of unrestricted eating may have contributed to inconsistent results in hormonal outcomes. While the fasting days were controlled, the absence of strict dietary restrictions during the free eating days could have influenced the stability of reproductive hormones, which are highly sensitive to dietary and metabolic fluctuations.

In addition, the control groups in this study, particularly the No Treatment Group and the Non-Meal Replacement Intermittent Fasting Group, offer valuable insights into the potential impact of the 5:2 intermittent fasting diet. The No Treatment Group helped account for natural fluctuations in body weight and metabolic markers, providing a baseline against which the effects of the fasting intervention could be assessed. This comparison revealed that the weight and metabolic improvements observed in the intervention group were greater than those in the no-treatment control, suggesting that the fasting intervention played a key role in these improvements. On the other hand, the Non-Meal Replacement Intermittent Fasting Group, which followed the same fasting regimen without meal replacements, allowed us to better isolate the effect of the meal replacement component. This group showed moderate improvements in weight and metabolic markers, but not as significant as those observed in the intervention group. This suggests that the inclusion of meal replacements likely enhanced the efficacy of the 5:2 intermittent fasting diet. However, the free eating days contributed to variability in dietary exposure across the study period, which may have made it difficult to attribute the outcomes solely to fasting. Therefore, while the improvements in body weight, insulin resistance, and metabolic markers are encouraging, the unchanged reproductive markers highlight the complexity of PCOS treatment. It suggests that more comprehensive or longer-term interventions, possibly combining intermittent fasting with other targeted therapies (e.g., anti-androgens or ovulation induction), may be required to see significant changes in reproductive hormone levels. Future studies with larger cohorts and longer durations are needed to explore the full spectrum of the effects of intermittent fasting on reproductive health in PCOS patients.

Previous studies have mentioned that overweight or obese PCOS patients typically have elevated FPG and FIN (5). FIN interferes with the secretion of LH and FSH by affecting the function of the hypothalamic–pituitary–ovarian axis, stimulates the production of androgens by the ovary and adrenal gland, reduces the synthesis and secretion of sex hormone-binding globulin (SHBG) in the liver, and increases the level of free testosterone in the blood. It affects follicle development and maturation disorders, leading to anovulatory infertility (26). Therefore, obesity, hyperandrogenism, and insulin resistance may be interrelated factors.

Compared with time-restricted feeding (TRF) and other intermittent fasting formats, the 5:2 MR approach has some distinct features. TRF (daily fasting window) has shown mixed results: one trial of 16h daily fast (5–6 weeks) in anovulatory PCOS achieved weight loss and significant drops in insulin, insulin resistance and androgen markers (27), whereas a recent randomized study of early-TRF (14:10) found no greater benefit on blood pressure or CRP than a standard diet (28).

The 5:2 intermittent fasting diet with meal replacement (MR) provides a strategy that includes two non-consecutive fasting days per week, where participants consume only 500 kcal per day. This approach may be easier for some patients to follow compared to daily calorie restriction, as it limits the fasting days to just two per week while ensuring adequate nutrient intake through the meal replacement products on fasting days. In contrast, alternate-day fasting, a similar intermittent fasting protocol, has been shown to produce slightly more weight loss, but adherence can be more challenging (29).

Our study demonstrated that the 5:2 intermittent fasting diet with MR significantly reduced body weight and improved insulin resistance, aligning with results from continuous calorie restriction (CR) interventions. The intermittent fasting approach with MR provides a flexible metabolic model, where 25% of days involve near-fasting, allowing for metabolic flexibility while reducing the risk of diet fatigue. Although this study did not explicitly compare the 5:2 intermittent fasting with MR to other regimens, its adherence rate (~75% completion) and significant findings suggest that this protocol is a viable option for managing weight and metabolic health in obese PCOS patients.

It is important to note that this study utilized specific commercial MR products, namely Kang Zhijun meal packs, which may affect the reproducibility of the findings. While Kang Zhijun MR products offer a controlled nutrient profile, the variability in ingredients and formulation across different brands could potentially lead to different metabolic or hormonal effects. Therefore, it is important to acknowledge that the results observed in this study may not be fully generalizable to other MR products, and future studies should consider comparing different MR formulations to assess whether the specific ingredients or composition of the meal replacement products impact the outcomes, particularly in PCOS patients.

It is important to note that this study adds valuable data on the effects of intermittent fasting in a Chinese patient population, which is underrepresented in the existing PCOS fasting literature. Additionally, the use of locally available meal replacement products, such as Kang Zhijun meal packs, provides insight into the feasibility of implementing this intervention in an Asian context. However, the generalizability of these findings to other regions or meal replacement products may require further exploration.

Several limitations of this study should be acknowledged. First, the sample size was limited, and some participants withdrew from the study due to poor compliance. As a result, no definitive conclusions can be made regarding the impact of the 5:2 intermittent fasting diet with MR on reproductive hormone levels, and further studies with larger sample sizes are necessary. Second, the 12-week duration of the intervention was relatively short, and long-term effects, as well as patient adherence over time, need to be validated in future research. Moreover, long-term follow-up data on the sustained impact of the intervention are essential to determine its efficacy and sustainability. Additionally, as a non-randomized, single-arm study, it is important to acknowledge that there is an inherent risk of selection bias and reporting bias due to the lack of randomization and blinding. These biases could potentially influence the study’s outcomes and interpretations. Although the study adhered to ethical guidelines under the 1964 Helsinki Declaration and obtained informed consent, future studies may benefit from randomization and blinding to better control for these potential biases. Furthermore, the inclusion of five unrestricted eating days in the 5:2 intermittent fasting regimen may have introduced dietary variability, which could influence the consistency of metabolic and hormonal responses. While this design reflects a real-world approach to improve adherence, it may also obscure the isolated effects of the fasting intervention itself. Future studies incorporating more structured dietary guidance during non-fasting days, or longer intervention periods, may help to better delineate the specific contributions of intermittent fasting versus overall dietary pattern changes.

In conclusion, this study found that 12 weeks of 5:2 intermittent fasting with MR can effectively reduce weight and improve metabolic markers in obese PCOS patients. Given its positive outcomes, the 5:2 intermittent fasting diet with MR could serve as an effective initial lifestyle intervention for obese women with PCOS. Further research with larger cohorts and extended follow-up periods is needed to confirm the long-term benefits and potential for improving reproductive health outcomes in this population.

## Data Availability

The original contributions presented in the study are included in the article/**Supplementary Material**. Further inquiries can be directed to the corresponding author.
