# Supplementary material for: Effect of a 5:2 intermittent fasting diet on obese patients with polycystic ovary syndrome
Source: Front Endocrinol (Lausanne). 2026 Mar 18;17:1758805. doi: 10.3389/fendo.2026.1758805 (PMC13038506; doi:10.3389/fendo.2026.1758805)
Supplement: Supplementary file 1 [file Table1.docx]

Supplementary table1 Nutrition Facts of Kang Zhijun™

|  | Package A | Package B |
| --- | --- | --- |
| Calories | 94kcal per serving | 88kcal per serving |
| Weight | 20g per serving | 20g per serving |
| Protein | 17.2g per100g; 29% | 19.7g per 100g; 33% |
| Fat | 19g per 100g; 32% | 11.4g per 100g; 19% |
| Total carbohydrates | 57.6g per 100g; 19% | 63.4g per 100g; 21% |
| Sodium | 368mg per 100g | 349mg per 100g |
| Ingredients | Medium-chain triglycerides, extra virgin olive oil, sea buckthorn oil, DHA algal oil, maltodextrin, fructo-oligosaccharides, whole milk powder, soy protein powder, inulin, xylitol, sweet orange powder, cheese powder, calcium (calcium carbonate), iron (ferric pyrophosphate), zinc (zinc oxide), vitamin A, vitamin D, vitamin E, vitamin B1,vitamin B2,vitamin B6,vitamin B12, vitamin C, nicotinic acid, folic acid, food additives (casein sodium, monoglyceride and diglyceride fatty acid esters, sucralose) | Grain flour (rice, soybeans, beer malt, oats, brown rice), xylitol, inulin, soy protein isolate, resistant dextrin, conjugated linoleic acid type edible vegetable oil, whey protein, kudzu powder, Chinese yam powder, goji berry powder, white kidney bean powder, food additives (casein sodium, monoglyceride and diglyceride fatty acid esters, sucralose) |

a One serving of Kang zhijun™ A (20g, 94 kcal, containing 11.5gcarbohydrates, 3.4g protein, and 3.8gfat) was dissolved in 200ml of water to replace breakfast, lunch, and dinner.

b For the remaining five days of the week, patients followed theirregular diet for breakfast and lunch, and used one serving of Kang zhijun™ B (20gdissolved in 200ml of water) to replacedinner. Each serving of meal powder B provided 88 kcal with 12.7g of carbohydrates, 3.9g of protein, and 2.3g of fat.
